# Archaeal Persisters: Persister Cell Formation as a Stress Response in *Haloferax volcanii*

**DOI:** 10.3389/fmicb.2017.01589

**Published:** 2017-08-21

**Authors:** Julianne Megaw, Brendan F. Gilmore

**Affiliations:** Biofilm Research Group, School of Pharmacy, Queen’s University Belfast Belfast, United Kingdom

**Keywords:** persister, stress response, haloarchaea, *Haloferax volcanii*, biofilm, quorum sensing

## Abstract

Persister cells are phenotypic variants within a microbial population, which are dormant and transiently tolerant to stress. Persistence has been studied extensively in bacteria, and in eukaryotes to a limited extent, however, it has never been observed in archaea. Using the model haloarchaeon, *Haloferax volcanii* DS2, we demonstrated persister cell formation in this domain, with time-kill curves exhibiting a characteristic biphasic pattern following starvation or exposure to lethal concentrations of various biocidal compounds. Repeated challenges of surviving cells showed that, as with bacteria, persister formation in *H. volcanii* was not heritable. Additionally, as previously shown with bacteria, persister formation in *H. volcanii* was suppressed by exogenous indole. The addition of spent culture media to assays conducted on planktonic cells showed that *H. volcanii*-conditioned media stimulated persistence, whereas conditioned media of other haloarchaea or halophilic bacteria did not, suggesting the involvement of a species-specific signal. Using a TLC overlay assay, the quorum sensing bioreporter *Agrobacterium tumefaciens* ATCC BAA-2240 detected the presence of C_4_ and C_6_ acyl homoserine lactone-like signal molecules in a *H. volcanii* culture extract. While synthetic bacterial AHLs did not induce persistence, this is potentially due to structural differences between bacterial and archaeal signals, and does not discount a quorum sensing component in haloarchaeal persister formation. The observation of persister cell formation by this haloarchaeon may provide some insights into the survival of these organisms in stressful or dynamic environments.

## Introduction

When microbial populations are exposed to stress which exceeds the capacity of genetically encoded protective responses, the majority are killed. Killing is commonly quantified by measuring the proportion of viable cells as a function of exposure time to the condition of interest, and generally exhibits a non-linear, biphasic killing pattern, with an initial exponential decline followed by a plateau or period of slower decline, with the latter part of the killing curve indicating the presence of a small subpopulation of specialized survivor cells, known as persisters (Balaban et al., 2004; Keren et al., 2004a). Kim and Wood (2016) proposed a working definition of persisters as “those cells that remain after the actively respiring cells are vanquished by antibiotics (usually seen as an unchanging or slowly decreasing concentration of viable cells)”.

Unlike resistant mutants, persisters are genetically identical to the cells which are killed, but are phenotypic variants which have entered a transiently quiescent state in which they are metabolically inactive, neither growing nor dying when exposed to lethal stress (Lewis, 2010). This process is reversible, therefore persisters act as a reservoir which, when favorable conditions are restored, gives rise to a new population of cells. The new population is as susceptible as the original, and possesses a similarly small proportion of persister cells; this differentiates persisters from resistant mutants, which are characterized by their stable and heritable insensitivity (Balaban et al., 2004; Keren et al., 2004a).

The phenomenon of persistence has been observed repeatedly since it was first demonstrated during an early study on the mechanism of action of penicillin by Hobby et al. (1942) that it was ineffective against non-growing *Staphylococcus* cells. This was later confirmed by the work of Bigger (1944), who showed that inhibiting the growth of *Staphylococcus* cultures by lowering the temperature, removing nutrients, or adding bacteriostatic concentrations of boric acid resulted in the generation of more persisters. More recently, Kwan et al. (2013) induced high levels of persistence (10–100%) from an initial population of 0.01% in *Escherichia coli* by halting transcription, translation, or ATP synthesis, using rifampin, tetracycline, and carbonyl cyanide *m*-chlorophenylhydrazone, respectively, confirming that persistence is the result of cells being in an inactive state.

While some previous explanations for the presence of persister cells is their stochastic generation in microbial populations (Balaban et al., 2004), or as a “bet-hedging strategy” ensuring the survival of the population in the event that lethal conditions should arise, their formation is now accepted to be a stress response, as persistence is not a random event and has frequently been shown to be induced by various environmental cues. Initially, it was demonstrated that maintaining a bacterial culture in early exponential phase by repeated dilution led to the elimination of persisters, and that the number of persister cells increased dramatically from mid-exponential to stationary phase, suggesting that persister formation was dependent on growth phase (Keren et al., 2004a). Although mainly studied with regard to antibiotic treatment, it has now been proposed that exposure to any stress condition could induce the formation of surviving persisters (Lewis, 2010). In addition to antibiotic exposure (Dörr et al., 2010; Kwan et al., 2013; Van den Bergh et al., 2016), persister formation has been shown to be stimulated by starvation (Maisonneuve et al., 2013), carbon source transitions (Amato et al., 2013), oxidative stress (Wu et al., 2012), toxic metals (Harrison et al., 2007), quorum sensing (Möker et al., 2010), host macrophages (Helaine et al., 2014), and the SOS response (Bernier et al., 2013). Additionally, persisters are not found in early phase microbial cultures, with any reports of persisters in early phase cultures assumed to be a result of carry-over from a stationary phase inoculum (Keren et al., 2004a). Gene pairs known as toxin–antitoxin (TA) systems, comprising a toxin and cognate antitoxin, have generally been accepted to be effectors and modulators of persistence. During normal growth conditions, the activity of toxins are neutralized by the antitoxins, but under conditions of stress, the antitoxins are degraded, enabling the toxins to exert their effects, which involve corruption of an essential cellular process, such as DNA replication or protein translation, leading to growth arrest and dormancy (Keren et al., 2004b; Shah et al., 2006; Page and Peti, 2016). A study by Chowdhury et al. (2016) also showed that the alarmone guanosine tetraphosphate (ppGpp), a key metabolite commonly linked to persistence, was not in fact required for persister formation in *E. coli*. These findings suggest that there are further, less explored or as yet unknown, modulators of persistence.

Previous research on persisters has almost exclusively been in a clinical context, focusing on the tenacity of pathogenic bacteria following antibiotic treatment. Persisters have been shown to have a major role in recurrent or chronic bacterial infections (Lewis, 2010; Fauvart et al., 2011), but it is likely that they also play a role in the survival and preservation of natural microbial populations, and it has been suggested that these dormant cells can act as a “seed bank,” enabling populations to recover and repopulate their environment following a catastrophic event (Lennon and Jones, 2011). The environments inhabited by haloarchaea are both extreme and dynamic, and consequently, they are exposed to stressful conditions including fluctuations in salinity (Becker et al., 2014; Thombre et al., 2016), intense solar radiation (Baliga et al., 2004; McCready et al., 2005), low dissolved oxygen (Grant, 2004), pH extremes (Mesbah et al., 2007; Mormile et al., 2009), and even entrapment for millennia (Grant et al., 1998; McGenity et al., 2000; Stan-Lotter et al., 2002; Lowenstein et al., 2011). In any dynamic environment, or when faced with sudden or transient stress, the phenotypic plasticity of microbial populations may be key in ensuring their survival. Published research has shown that archaeal genomes contain toxin–antitoxin loci (Gerdes et al., 2005; Pandey and Gerdes, 2005), suggesting a capacity for persister cell formation, but none as yet have provided experimental evidence to demonstrate the presence of the phenotype in this domain. Using the model haloarchaeon, *Haloferax volcanii* DS2, this study aimed to investigate whether persister cell formation is a strategy employed by haloarchaeal populations to withstand and recover from lethal stress.

## Materials and Methods

### Growth Medium and Conditions

*Haloferax volcanii* DS2 (DSM 3757) was obtained from DSMZ (Braunschweig, Germany) and used for all experiments. *H. volcanii* was grown and maintained aerobically at 37°C in broth containing, in g L^-1^, NaCl (150), MgSO_4_.7H_2_O (20), KCl (2), FeSO_4_.4H_2_O (0.036), MnCl_2_.4H_2_O (3.6 × 10^-3^), yeast extract (10), casein hydrolysate (7.5), trisodium citrate (3), at pH 7.4, with 1.5% agar added for solid media.

### Antimicrobial Susceptibility Testing

Sterile aqueous stock solutions of the biocides H_2_O_2_, NaClO, 1-dodecyl-3-methylimidazolium chloride ([C_12_mim]Cl), chlorhexidine, and the antibiotic rifampicin were used to prepare minimum inhibitory concentration (MIC) assays. Serial doubling dilutions of each biocide (six replicates) were prepared in 100 μL sterile broth in 96-well microtitre plates. An inoculum was prepared by adjusting the turbidity of an exponential phase broth culture of *H. volcanii* to correspond with a cell density of 2 × 10^5^ cfu mL^-1^, which was verified by total viable count. 100 μL was added to each well of the microtitre plates. Positive and negative growth controls were also included in each plate. Plates were incubated at 37°C until copious growth was evident in the positive control wells, and MIC values were determined for each compound as the lowest concentration at which growth was inhibited. Minimum biocidal concentrations (MBC) were determined by inoculating plates of the above medium with 20 μL spots of broth from clear wells and incubating at 37°C for 5 days, and were defined as the lowest concentration which reduced the initial inoculum by >99.9%, as determined by total viable counts.

### Persister Cell Assays

The OD_590_ of a stationary phase liquid culture of *H. volcanii* DS2 was adjusted to 0.1 in sterile broth. For the biocides, 10 mL volumes of the cell suspension were transferred to 50 mL microcentrifuge tubes, and MBC concentrations of each of the chosen compounds (0.005% H_2_O_2_, 5 μg mL^-1^ NaClO, 2 μg mL^-1^ [C_12_mim]Cl, 10 μg mL^-1^ chlorhexidine, and 50 μg mL^-1^ rifampicin) were added (three replicates). For the starvation assay, 10 mL samples of the cell suspensions were centrifuged, the broth discarded, and the pellets resuspended in 10 mL sterile 15% NaCl. All tubes were incubated at 37°C and at specific time intervals, three 200 μL samples were taken and serially diluted to obtain total viable counts for each time point, and cell viability expressed as a percentage of Time 0 samples. To determine persister heritability, colonies were picked from plates showing growth following the persister assays, re-grown in broth to an OD_590_ of 1.0, and the assays performed again as above.

### Persister Cell Formation in Biofilms

Biofilms of *H. volcanii* were grown using the MBEC device (Innovotech). The OD_590_ of a *H. volcanii* liquid culture was adjusted to 0.1, and 150 μL was inoculated into each well of the plate supplied with the device, which was then incubated for 3 weeks at 37°C, with the lid being placed weekly into a new plate containing fresh broth. Following incubation, three pegs were removed using sterilized pliers for each condition to be tested. The pegs were rinsed by placing into the wells of a 96-well plate containing 200 μL sterile 15% NaCl to remove any loosely adhered or planktonic cells. The pegs were then placed in 200 μL of broth containing MBC concentrations of H_2_O_2_, NaClO, and chlorhexidine in a 96-well microtitre plate. Following incubation for 6 h, the pegs were placed in the top row of a 96-well plate containing 200 μL 15% NaCl in the top row, and 180 μL in the other rows. The plate was sonicated in an ultrasonic bath for 30 min, the pegs discarded, and serial dilutions were performed to obtain total viable counts, expressed in cfu peg^-1^. Plates were incubated at 37°C for 5 days and counts were expressed for each treatment as a percentage of those obtained for untreated pegs.

### Conditioned Media

A stationary phase liquid culture of *H. volcanii* was centrifuged and the pellet resuspended in broth containing double concentrations of yeast extract and casein hydrolysate to an OD_590_ of 0.2. 5 mL aliquots of this suspension were added to 50 mL centrifuge tubes, along with 5 mL sterile filtered spent broth from stationary phase cultures of *H. volcanii*, other haloarchaea (*Halococcus dombrowskii*, *Halorubrum trapanicum*, and *Halobacterium noricense*), or halophilic bacteria (*Halomonas janggokensis*, *Chromohalobacter canadensis*, and *Staphylococcus succinus*), from our own strain library of isolates from a Triassic salt mine. A control with fresh, sterile broth added was also included. All tubes were incubated at 37°C for 1 h, then H_2_O_2_ was added to a final concentration of 0.005%. Viable counts were obtained following incubation for 6 h and were expressed as a percentage of counts obtained at Time 0.

### Effect of Indole on Persister Cell Formation

The persister assay was conducted with 0.005% H_2_O_2_ as before, except prior to challenge, cell suspensions were incubated at 37°C for 1 h, either in the presence or absence of 500 μM indole. The percentage of survivors at 6 h was compared with and without indole.

### Determination of Acyl Homoserine Lactone Production by *H. volcanii* DS2

A stationary phase culture of *H. volcanii* was extracted with three volumes of EtOAc by stirring overnight. The EtOAc layer was removed and dried under reduced pressure, weighed, and then reconstituted in EtOAc to create a 50 mg mL^-1^ stock solution. 0.25 mg of the extract was run on a C_18_ reversed-phase TLC plate, along with standards of *N*-butyryl, -hexanoyl -octanoyl (1 μL of 100 μM), and -dodecanoyl (1 μL of 10 mM) -DL-homoserine lactones (Sigma-Aldrich, United Kingdom), using a mobile phase of 55:45:0.001 MeOH:H_2_O:CH_3_COOH. *Agrobacterium tumefaciens* ATCC BAA-2240 was grown in LBB with 30 μg mL^-1^ gentamicin overnight at 28°C. Glass tubes containing 10 mL LB with 0.5% agar were boiled to liquefy the agar and then cooled to 55°C. 80 μL of a 10 mg mL^-1^ X-gal solution and 1 mL *A. tumefaciens* broth culture were added to each tube. The dried TLC plates were overlaid with the molten agar in a sterile Petri dish and incubated at 28°C.

### Effect of Acyl Homoserine Lactones on Persister Cell Formation

Synthetic acyl homoserine lactones (*N*-butyryl, -hexanoyl -octanoyl and -dodecanoyl -DL-homoserine lactone) were added to 10 mL samples of an early stage broth culture of *H. volcanii* DS2 (OD_590_ 0.1), to a final concentration of 10 μM, and incubated for 1 h. A control with no AHL was also included. 0.005% H_2_O_2_ was added and total viable counts were obtained after 6 h, and expressed as a percentage of Time 0 values.

### Identification of Toxin–Antitoxin Loci

The complete sequences of the genome of *H. volcanii* DS2 and its four plasmids were downloaded from NCBI GenBank. Toxin–antitoxin loci were identified using TAfinder^1^ using the default parameters (Shao et al., 2011).

### Statistical Analysis

Statistical analyses were performed using GraphPad Prism 6. All values in figures are expressed as the mean of three replicates ± one standard deviation. Kruskal–Wallis one-way analysis of variance (ANOVA) with Dunn’s *post hoc* multiple comparisons test was used to analyze statistical differences between test samples and controls; a paired *t*-test (two-tailed) was used to analyze differences between pairs of data. For all analyses, a value of *p* < 0.05 was considered significant.

## Results and Discussion

### Persister Cell Formation by *Haloferax volcanii*

**FIGURE 1:**
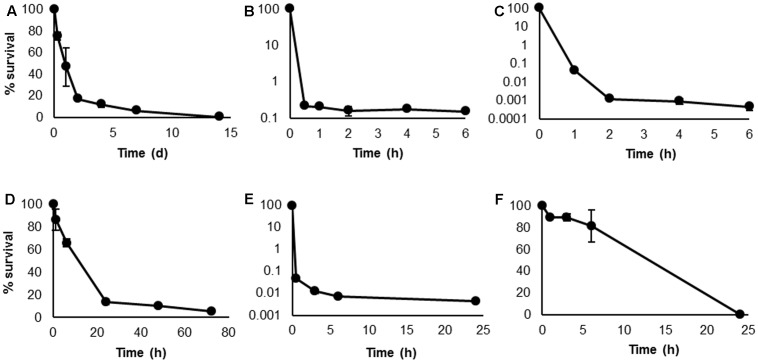
Kill curves of *Haloferax volcanii* following biocidal treatments. Survival was determined by total viable counts at specific time points, under conditions of starvation **(A)**, 0.005% H_2_O_2_ **(B)**, 5 μg mL^-1^ NaClO **(C)**, 2 μg mL^-1^ [C_12_mim]Cl **(D)**, 10 μg mL^-1^ chlorhexidine **(E)**, and 50 μg mL^-1^ rifampicin **(F)**. Plotted values are the mean of triplicate measurements and error bars represent ± SD.

Persister cell formation by *H. volcanii* was determined based on total viable counts at specific time points, and under the conditions of starvation, and treatment with biocidal concentrations of H_2_O_2_, NaClO, [C_12_mim]Cl, and chlorhexidine, a characteristic, biphasic killing pattern was observed, with a rapid initial decline followed by a distinct plateau indicative of small surviving persister subpopulations (**Figures 1A–E**). This killing pattern is the definitive indicator of persister formation (Keren et al., 2004a; Maisonneuve and Gerdes, 2014), and provides confirmation of this phenotype in the domain Archaea.

The majority of studies on persister cells are conducted using clinically relevant bacteria and focus on their response to antibiotic treatment. However, as the focus of this study was an environmental haloarchaeal species with no clinical relevance and susceptibility to few antibiotics, this approach is less appropriate. We therefore chose stimuli which may be more relevant to haloarchaea: starvation, oxidative stress induced by H_2_O_2_ and NaClO, and membrane disruption induced by [C_12_mim]Cl and chlorhexidine. Rifampicin was also selected as it is one of the few antibiotics to which most haloarchaeal genera show susceptibility (Bonelo et al., 1984). In contrast to the other treatments, rifampicin-treated cells did not exhibit a logarithmic killing pattern, but a steady, linear decline until survival reached 0% (**Figure 1F**). As the other treatments indicated the presence of persisters in this species, it appears that rifampicin eradicated the persisters in addition to the bulk population of cells. Recently, rifampicin has been used as a pre-treatment in bacterial studies to convert exponentially growing cells to the persister phenotype (Kwan et al., 2013, 2015a; Lee et al., 2016). However, the effects of rifampicin on bacteria and archaea are not comparable. In bacteria, the mechanism of action of rifampicin is inhibition of DNA-dependent RNA polymerase, while in archaea, rifampicin induces cell lysis by exerting a detergent effect on the cell membrane (Pfeifer, 1988). Based on the results obtained here, it is apparent that rifampicin is destructive to all *H. volcanii* cells, including persisters.

**FIGURE 2:**
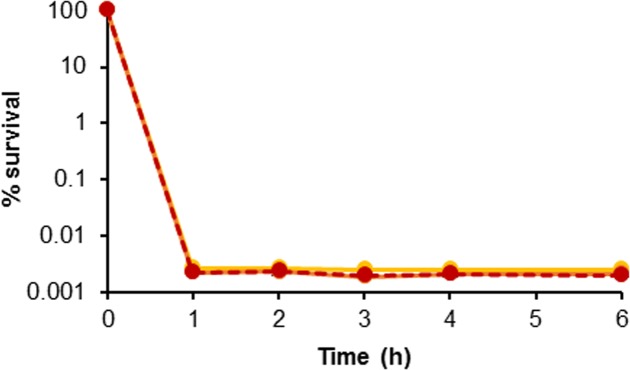
Evaluation of persister cell heritability. Kill curves of *H. volcanii* following repeated challenges of surviving colonies in 0.005% H_2_O_2_. Yellow, orange, and red lines are replicate 1, 2, and 3, respectively. Plotted values are the mean of triplicate measurements and error bars represent ± SD.

The second major identifier of persistence is non-heritability of the trait (Keren et al., 2004a). In order to demonstrate that the surviving population was a result of the phenotypic response of persister cell formation and not genetically acquired tolerance or resistance, colonies were picked from the plates on which surviving cells were enumerated; these were grown as broth cultures and were tested again as before. **Figure 2** shows a challenge with H_2_O_2_ repeated three times. No inherited resistance was observed following repeated challenges of surviving cells, as three almost-identical biphasic kill curves were obtained. This confirms that persistence in *H. volcanii* is a non-heritable phenotypic trait, as enrichment or increase in the surviving population was not possible, and indicates that the survival mechanism is likely similar to the phenotypic response frequently described for bacteria.

### Persister Cells in *H. volcanii* Biofilms

**FIGURE 3:**
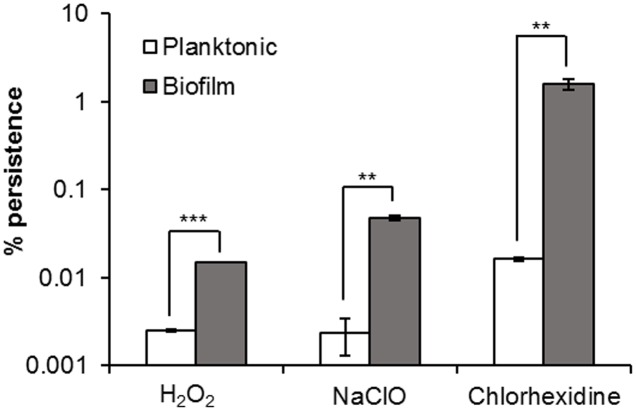
Persister cells in *H. volcanii* biofilms. Comparison of percentage survival of planktonic and biofilm cultures of *H. volcanii*, following incubation for 6 h in biocidal concentrations of H_2_O_2_, NaClO, and chlorhexidine. Plotted values are the mean of triplicate measurements and error bars represent ± SD. Asterisks denote significance values as determined by paired *t*-tests: ^∗^*p* < 0.05; ^∗∗^*p* < 0.01; ^∗∗∗^*p* < 0.001.

Considerably greater proportions of persister cells were observed in *H. volcanii* biofilms in comparison to stationary phase planktonic cultures for the three biocides tested. Challenge with H_2_O_2_ resulted in 0.0025 and 0.015% survival in planktonic culture and biofilm respectively, with NaClO resulting in 0.002 and 0.047%, respectively, and chlorhexidine 0.016 and 1.6% respectively (**Figure 3**). Six hours was deemed to be an appropriate time point to determine persister numbers, as based on the kill curves shown in **Figure 1**, for the three biocides, they had all unquestionably reached a plateau by this time. For cells challenged with H_2_O_2_, there was little to no variation in cell number after 30 min–1 h, for NaClO, 2 h, and for chlorhexidine, 6 h. Taking data from studies on bacteria into consideration, the marked difference in persister cells between planktonic and biofilm cultures is not what would be expected, as it has been shown that stationary phase planktonic cultures and biofilms should contain a similar proportion of persisters (Spoering and Lewis, 2001; Lewis, 2008). Haloarchaeal biofilms were only recently described and investigated (Fröls et al., 2012), and while biofilms of *H. volcanii* DS2 have been shown to have many phenotypic features in common with bacterial biofilms, they also appear to possess some unique features (Chimileski et al., 2014). As haloarchaeal biofilms are currently not well-studied in comparison to bacterial biofilms, it is unclear whether they should be expected to behave in the same manner.

Spoering and Lewis (2001) suggested that the maintenance of a subpopulation of persister cells is strongly dependent on the population density, and that the common method of conducting assays on diluted stationary phase cultures leads to a collapse in the number of persisters. This indicates that the dense populations of biofilms or stationary phase cultures favors persister formation, and that the common misconception that biofilms are more resistant to killing, or have more persisters than planktonic cultures, derives from experiments using either logarithmic or diluted stationary phase cultures, and are therefore based upon a false comparison, while a more direct comparison should be made between biofilms and undiluted stationary phase cultures. As diluted stationary phase cultures were used for planktonic cell assays, this could provide a reason for the difference in persister cell number, however, as the cell suspensions were treated immediately following dilution, this seems unlikely to be the sole explanation. It is known that extended incubation of diluted stationary phase cultures prior to treatment will cause some persisters to revert and become sensitive. However, the rate of resuscitation of dormant cells appears to be dependent on various factors, such as the composition of the medium into which they are diluted, and the time between dilution and challenge (Jõers et al., 2010). Additionally, Kim et al. (2011) showed that reversion of *E. coli* and *Pseudomonas aeruginosa* persisters to normal cells was not instantaneous but took several hours. The slow growth rate of haloarchaea in comparison to fast-growing bacterial species such as *E. coli*, and their immediate challenge following dilution make dilution even less likely to have been a factor. It could transpire that this difference is the result of an as-yet-unknown feature of haloarchaeal biofilms favoring persister cell formation.

### Influence of the Interkingdom Signal Indole on Persistence

**FIGURE 4:**
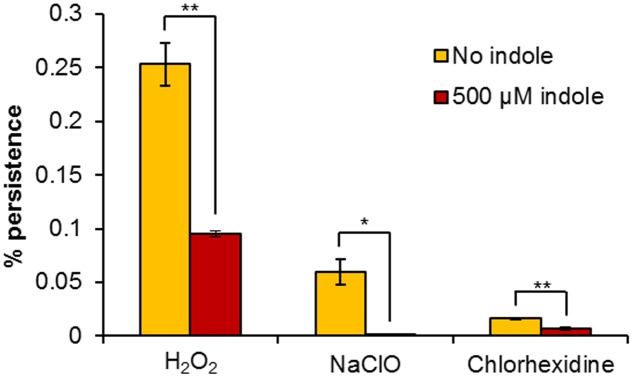
Influence of exogenous indole on *H. volcanii* persistence. Percentage survival of *H. volcanii* pre-incubated for 1 h with or without 500 μM indole, prior to 6 h challenge with biocidal concentrations of H_2_O_2_, NaClO, and chlorhexidine. Plotted values are the mean of triplicate measurements and error bars represent ± SD. Asterisks denote significance values as determined by paired *t*-tests: ^∗^*p* < 0.05; ^∗∗^*p* < 0.01; ^∗∗∗^*p* < 0.001.

Indole is known as an interspecies and interkingdom signaling molecule with numerous biological roles in microbial communities, including spore formation, plasmid stability, drug resistance, biofilm formation, and virulence among species which produce indole, and even in some cases, those which do not (Lee and Lee, 2010). Vega et al. (2012) showed that incubation of *E. coli* cells for 1 h in 500 μM indole prior to an antibiotic challenge resulted in a large increase in persister cell formation, which was attributed to the induction of oxidative stress and phage-shock responses, suggesting that indole signaling has a role in the stimulation of persister cell formation. Subsequent studies have shown that indole does have a role in persistence, but it is more likely to be involved in suppression. We conducted our study under the same conditions (incubation with 500 μM indole for 1 h prior to treatment). We also confirmed that the concentration of indole used had no effect on the growth and survival of *H. volcanii*, with an MIC value in excess of 2 mM. Pre-incubation of *H. volcanii* cell suspensions with indole prior to challenge with biocidal concentrations of H_2_O_2_, NaClO, or chlorhexidine resulted in 2.7-, 188-, and 2.3-fold decreases respectively in persister cell formation in comparison to cells which were not pre-treated. While two of these decreases were very small, they were statistically significant (**Figure 4**). These results indicate that indole appears to reduce the proportion of persister cells in *H. volcanii*, which is in agreement with more recent studies showing an inversely proportional relationship between indole concentration and persistence. Hu et al. (2015) showed that the toxin YafQ increases persister cell formation in *E. coli* by cleaving tryptophanase (TnaA) mRNA and inhibiting the ability of cells to produce indole, TnaA deletion decreased persistence, and that the addition of exogenous indole reduced persister formation, as did the addition of tryptophan. Kwan et al. (2015b) showed that production of DosP (direct oxygen sensing phosphodiesterase) increased persistence in *E. coli* by degrading another cyclic nucleotide signal, cyclic adenosine monophosphate (cAMP), which results in lower indole levels due to reduced TnaA activity. Lee et al. (2016) showed that indole and modified indoles reduced persisters in *E. coli* and *Staphylococcus aureus*, or even eradicated them completely. It has been previously reported that *H. volcanii* tryptophanase functions as an inducible promoter in this species, with expression increasing rapidly following the addition of tryptophan to the medium (Large et al., 2007). Further research could explore the role of tryptophan and tryptophan-inducible gene expression in persister formation in *H. volcanii*.

### Toxin–Antitoxin Loci in *H. volcanii*

Searching the genome and plasmid sequences of *H. volcanii* in TAfinder highlighted some putative TA loci (**Table 1**). The TA system with the highest sequence similarity to known TA genes contained a PIN domain (nuclease) toxin and RHH domain antitoxin, with a match of 84.01%. Also highlighted were the common prokaryotic *vapBC* system (65.84%), the translation repressors RelE with an RHH antitoxin (53.55%), and HicAB (49.01%). Archaea have previously been shown to possess TA systems (Pandey and Gerdes, 2005; Yamaguchi et al., 2011), and the toxin RelE and the antitoxin RelB from the hyperthermophilic archaeon *Pyrococcus horikoshii* were structurally elucidated and shown to be homologous but with numerous differences to those found in *E. coli* (Takagi et al., 2005), so it is possible that haloarchaeal toxins and antitoxins are considerably divergent to those which are known. While TA systems are known to be important factors in the archaeal stress response – deletion of a specific *vapBC* locus in the thermophilic archaeon *Sulfolobus solfataricus* rendered it heat shock labile (Cooper et al., 2009), there is no current knowledge regarding their role in archaeal persister cell formation. Further work is required to determine the role of these loci and indeed whether the effect of indole addition resulted in their corruption, as has been shown previously.

**Table 1:** Toxin–antitoxin loci identified on *Haloferax volcanii* DS2 chromosome and plasmids.

TA no.	T/A	Location	Length (aa)	Strand	Family	Domain	T score/A score	TA score
								
**Chromosome (NC_013967.1)**
1	T	180588–180998	136	-		PRK09831	10.98	7.1
	A	180124–180504	126	-		pfam12840	1.29	
2	T	237887–237988	33	+	HicA-like domain		54.55	49.01
	A	237481–237741	86	+	HicB-like domain		40.7	
3	T	437012–437659	215	+	vapC		11.16	13.77
	A	437774–438166	130	+	ArsR-like domain		17.69	
4	T	1813914–1814372	152	+	vapC		71.05	65.84
	A	1813650–1813895	81	+	vapB		58.02	
								
**Plasmid pHV3 (NC_013964.1)**
1	T	81215–81886	223	-	vapC		12.56	12.31
	A	81961–82491	176	-	Xre-like domain		11.93	
								
**Plasmid pHV4 (NC_013966.1)**
1	T	189115–189552	145	-		cd09886	16.6	13.98
	A	189549–189824	91	-		COG3905	10.05	
2	T	379569–379850	93	-	relE-like domain	COG2026	54.07	53.55
	A	379843–380097	84	-	RHH-like domain	COG3609	52.76	
3	T	445961–446530	189	+	PIN-like domain		83.6	84.01
	A	445533–445964	143	+	RHH like domain		84.62	


### Influence of Conditioned Media on Persister Formation

**FIGURE 5:**
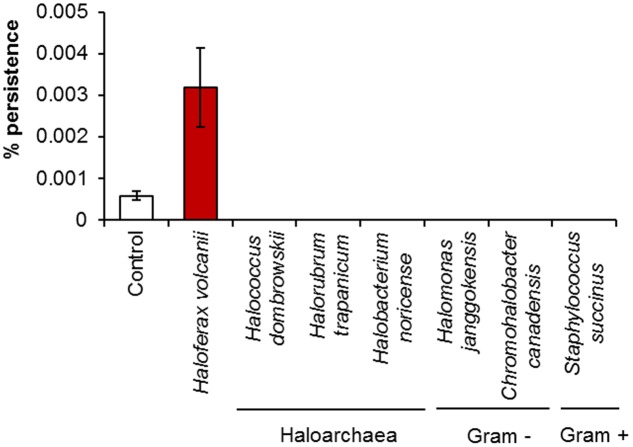
The effect of spent culture medium on persister cell formation by *H. volcanii*. Percentage survival of *H. volcanii* following incubation for 1 h in the presence of its own spent culture medium or the spent culture media of other haloarchaea or halophilic bacteria, followed by challenge for 6 h with 0.005% H_2_O_2_. Cells suspended in fresh broth served as a negative control. Plotted values are the mean of triplicate measurements and error bars represent ± SD.

When *H. volcanii* was challenged with 0.005% H_2_O_2_ in the presence of its own spent medium, it showed a fourfold increase in the number of persisters in comparison to fresh broth (**Figure 5**). It has already been established that persister cell formation is growth phase-dependent, increasing at late exponential to stationary phase (Keren et al., 2004a), and has been attributed to stress resulting from nutrient depletion, or increased cell signaling due to the higher population density. As the assays were conducted in 50% spent broth and 50% fresh broth with double concentrations of nutrients, nutrient levels in the assay conditions should have been comparable to fresh medium, which suggests that in this case, the elevated level of persistence was not induced by nutrient depletion during the pre-incubation period. No such increase in persistence was observed when the assay was conducted in spent media of other halophilic species (other haloarchaea plus Gram-positive and Gram-negative bacteria), all of which resulted in 0% survival after 6 h, which may suggest that a species-specific signal is involved in the induction of persistence. In addition to possibly not containing the specific signal which may be required, the spent media of the other species may also have contained compounds such as halocins or bacteriocins, or some other non-specific antimicrobial metabolite resulting in the complete eradication of the cells. Although differences were not statistically significant (*p* > 0.05) as determined by Kruskal–Wallis ANOVA, it is still apparent that the addition of conditioned media impacted persister formation in this species. The addition of spent growth medium has previously been shown to stimulate a significant increase in persister cell formation in *P. aeruginosa* PA14, and was attributed to the presence of various signaling molecules present in the medium. The same effect was not seen when the spent medium of *P. aeruginosa* PA14 was added to *E. coli* or *S. aureus* cultures, however, these species were also unaffected by the addition of their own spent medium (Möker et al., 2010). This suggests that persister cell formation is a response to a specific signal in some species, but the precise mechanisms for others may be different.

### Acyl Homoserine Lactone Production by *H. volcanii*

**FIGURE 6:**
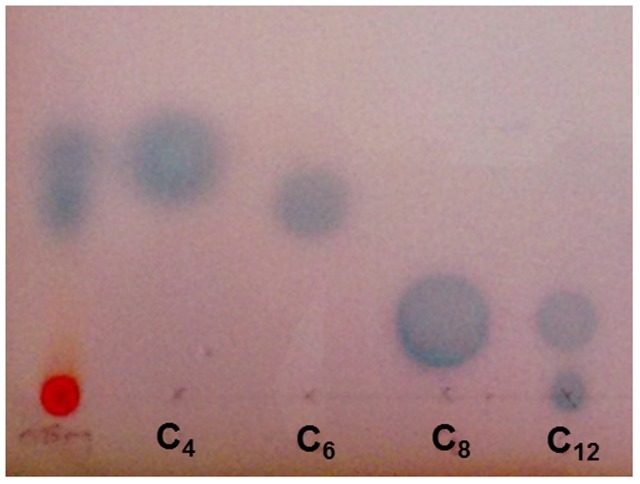
Acyl homoserine lactone production by *H. volcanii.* TLC overlay with *Agrobacterium tumefaciens* ATCC BAA-2240 showing two zones of activation in response to 0.25 mg EtOAc extract of *H. volcanii*, alongside standards of *N*-butyryl, -hexanoyl -octanoyl and -dodecanoyl -DL -homoserine lactone molecules.

The EtOAc extract of *H. volcanii*, when separated by TLC and overlaid with the bacterial biosensor *A. tumefaciens* ATCC BAA-2240, showed activation in two locations, which corresponded with the C_4_ and C_6_ AHL standards (**Figure 6**), suggesting the production of these two molecules by this haloarchaeon. Quorum sensing has been studied previously in archaea, but to a very limited extent. *A. tumefaciens* has previously been reported to detect quorum sensing signals from the haloalkaliphilic archaeon *Natronococcus occultus* (Paggi et al., 2003). Additionally, the methanogenic archaeon *Methanosaeta harundinacea* was shown to produce modified AHL molecules with an additional carboxyl moiety on the N atom of the homoserine lactone ring. These carboxylated AHLs induced a change in cellular morphology from short to filamentous, with Gram negative bacterial AHL molecules failing to induce the same effect (Zhang et al., 2012). While the mechanism of AHL production in archaea does not appear to be the same as in bacteria – they are not known to possess LuxI or LuxR genes – it was suggested that FilI, a histidine kinase, is an AHL synthase in this species. It has also been shown that diketopiperazines produced by the halophilic archaeon *Haloterrigena hispanica* could activate AHL bioreporters (Tommonaro et al., 2012). However, the spots produced on the TLC by the *H. volcanii* extract aligned with AHL standards, suggesting that they are in fact AHLs or AHL-like molecules. The extract did not activate the more specific AHL bioreporter *Chromobacterium violaceum* CV026 (not shown), suggesting that, while the signaling molecules produced are certainly AHL-like, they may not be identical to those used by bacteria, and it is quite possible that these too are carboxylated or carry some other slight modification, and further study would be required to elucidate their structures.

**FIGURE 7:**
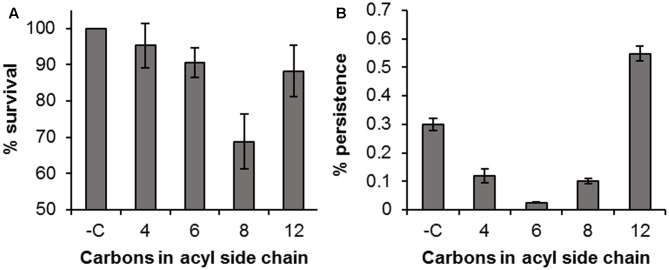
Antimicrobial activity of AHLs. Percentage survival of *H. volcanii* following incubation with 10 μM AHLs for 1 h compared to an untreated control (Time 0) **(A)**; percentage survival following treatment with 0.005% H_2_O_2_ in the presence of 10 μM AHLs, compared to the time 0 value **(B)**. Plotted values are the mean of triplicate measurements and error bars represent ± SD.

Pre-incubation of *H. volcanii* with exogenous AHL molecules prior to challenge showed that the AHLs themselves had a considerable antimicrobial activity against *H. volcanii*, with a reduction of 4.7, 9.4, 31.2, and 11.8% by C_4_, C_6_, C_8_, and C_12_ AHL respectively, in comparison to the untreated control (**Figure 7A**). This pattern was still evident, and more pronounced, following the 6 h treatment with H_2_O_2_ (**Figure 7B**). The observed killing pattern was characteristic of the biocidal activity of many classes of compounds bearing hydrophobic side chains (Birnie et al., 2000; Łuczak et al., 2010; Joondan et al., 2014), with activity increasing with chain length, up to a critical length after which it decreases, known as a “cut-off effect.” The results suggest that these AHLs have a biocidal activity against *H. volcanii*, with a chain length-dependent effect. The number of persisters in the samples incubated with C_12_ AHL prior to challenge was almost double that of the negative control, suggesting that this particular molecule may have stimulated persister formation, although this is unlikely to have been a quorum sensing effect as *H. volcanii* is not known to produce a C_12_ AHL, and is more likely a result of its antimicrobial activity.

It was shown by Zhang et al. (2012) that the quorum sensing controlled phenotype observed in *Methanosaeta harundinacea* could not be induced by bacterial AHLs. Although it appears that *H. volcanii* does produce AHL-like molecules, the exogenous molecules supplied may not have been sufficiently similar to its own, and not only did these molecules not induce the desired phenotype, they had a detrimental effect on cell viability. It has been shown previously that persister cell formation can be a quorum sensing controlled phenotype in bacteria. RelE-mediated dormancy in *E. coli* has been shown to be enhanced at high cell densities in comparison to low densities (Tashiro et al., 2012), quorum sensing based on acyl homoserine lactones or pyocyanin can induce persister formation in *P. aeruginosa* (Möker et al., 2010), and the stress-inducible quorum sensing CSP peptide can induce persister formation in *Streptococcus mutans* (Leung and Lévesque, 2012). Although not confirmed by this study, based on the results obtained using conditioned media, the possibility remains that persister formation in *H. volcanii* could still be a quorum sensing controlled phenotype. However, further study would be required to confirm whether or not the mechanism leading to persistence is AHL-based or controlled by another type of signal, and to determine the precise nature of the molecule or molecules responsible.

## Conclusion

Our results demonstrate the persister cell phenotype in Archaea. The formation of these specialized survivor cells has therefore now been observed in all three domains of life. Persister formation by haloarchaea could be a strategy employed by these organisms to ensure the survival of populations as a whole following exposure to lethal conditions, environmental fluctuations, or for long-term survival by inducing dormancy when adverse conditions are encountered. While further studies are required to determine the precise mechanisms of haloarchaeal persister formation, the role of toxin–antitoxin systems, indole signaling, and quorum sensing, this initial study may provide some insights into the survival of haloarchaea in stressful or dynamic environments.

## Author Contributions

BG and JM conceived and designed the study and experiments. JM conducted the experiments, analyzed the data, and wrote the manuscript. BG critically revised and approved the final manuscript.

## Conflict of Interest Statement

The authors declare that the research was conducted in the absence of any commercial or financial relationships that could be construed as a potential conflict of interest.

## Footnotes

1 http://202.120.12.133/TAfinder/index.php
